# Prevalence of enuresis and its impact in quality of life of patients with sickle cell disease

**DOI:** 10.1590/S1677-5538.IBJU.2019.0026

**Published:** 2019-01-29

**Authors:** Flávia Cristina de Carvalho Mrad, Alana de Medeiros Nelli, Mateus de Andrade Alvaia, Heros Aureliano Antunes da Silva Maia, Carina Oliveira Silva Guimarães, Evanilda Souza de Santana Carvalho, Cristiano Mendes Gomes, José Murillo Bastos Netto, José de Bessa

**Affiliations:** 1 Departamento de Cirurgia Universidade Estadual de Feira de Santana Feira de Santana BA Brasil Departamento de Cirurgia Universidade Estadual de Feira de Santana, Feira de Santana, BA, Brasil;; 2 Departamento de Pediatria Unidade de Nefrologia Pediátrica Universidade Federal de Minas Gerais Belo Horizonte MG Brasil Departamento de Pediatria, Unidade de Nefrologia Pediátrica, Universidade Federal de Minas Gerais, Belo Horizonte, MG, Brasil.;; 3 Programa de Pós-Graduação em Saúde Coeltiva Universidade Federal de Juiz de Fora Juiz de Fora MG Brasil Programa de Pós-Graduação em Saúde Coeltiva, Universidade Federal de Juiz de Fora, Juiz de Fora, MG, Brasil;; 4 Departamento de Cirurgia Faculdade de Ciências Médicas e da Saúde de Juiz de Fora Brasil Departamento de Cirurgia, Faculdade de Ciências Médicas e da Saúde de Juiz de Fora;Brasil; 5 Faculdade de Ciências Médicas e da Saúde de Juiz de Fora MG Brasil Faculdade de Ciências Médicas e da Saúde de Juiz de Fora, MG, Brasil;; 6 Hospital e Maternidade Terezinha de Jesus de Juiz de Fora Juiz de Fora MG Brasil Hospital e Maternidade Terezinha de Jesus de Juiz de Fora, Juiz de Fora, MG, Brasil

**Keywords:** Quality of Life, Sickle Cell Trait, Enuresis

## Abstract

**Introduction:**

Evidence indicates an increase in the prevalence of enuresis in individuals with sickle cell disease. The present study aims to evaluate the prevalence and impact of enuresis on quality of life in individuals with sickle cell disease.

**Materials and Methods:**

This cross-sectional study evaluated individuals with sickle cell disease followed at a reference clinic, using a questionnaire designed to evaluate the age of complete toilet training, the presence of enuresis and lower urinary tract, and the impact on quality of life of these individuals.

**Results:**

Fifty children presenting SCD (52% females, mean age ten years) were included in the study. Of those, 34% (17/50) presented as HbSC, 56% with HbSS (28/50), 2% Sα-thalassemia (1/5) and 8% the type of SCD was not determined. The prevalence of enuresis was 42% (21/50), affecting 75% of subjects at five years and about 15% of adolescents at 15 years of age. Enuresis was classified as monosymptomatic in 33.3% (7/21) and nonmonosymptomatic in 66.6% (14/21) of the cases, being primary in all subjects. Nocturia was identified in 24% (12/50), urgency in 20% (10/50) and daytime incontinence 10% (5/50) of the individuals. Enuresis had a significant impact on the quality of life of 67% of the individuals.

**Conclusion:**

Enuresis was highly prevalent among children with SCD, and continues to be prevalent throughout early adulthood, being more common in males. Primary nonmonosymptomatic enuresis was the most common type, and 2/3 of the study population had a low quality of life.

## INTRODUCTION

Sickle cell disease (SCD) is an autosomal recessive hereditary disease in which hemoglobin S is present (1, 2). According to the type of alteration present in hemoglobin, SCD can be classified in different clinical forms: homozygous form SS (referred to as sickle cell anemia-HbSS), and heterozygous forms, represented by associations of HbS with other hemoglobin defects ( SC, HbS/β0 thalassemia, HbS/β + thalassemia, S/α thalassemia) (1). SCD is the most common congenital hemoglobinopathy and affects mainly Africans or their descendants in America, being responsible for more than 300.000 live births per year (3, 4). In Brazil, 60.000 to 100.000 cases of the disease are currently estimated. SCD requires multi-professional approach for early diagnosis and management, due to its physical, psychological and socioeconomic impact, with high morbidity and mortality (1).

Lower urinary tract symptoms (LUTS) are also common in children occurring in about 14.7 to 21.8% (5, 6). LUTS is characterized by abnormal urine storage and/or bladder emptying in the absence of urinary tract infections, neurological or anatomical abnormalities (7, 8). Enuresis is both a symptom and a condition of intermittent incontinence that occurs during periods of sleep after the age of five years. According to symptoms, enuresis is classified as monosymptomatic, when no other symptom is present and non-monosymptomatic when associated with LUTS (8). Prevalence of enuresis in children aged 6 to 13 years varies from 9.5% to 12.9% (9, 10). Enuresis has adverse emotional and social effects that affect children’s quality of life (11).

The prevalence of enuresis in children with SCD is approximately 32% (12, 13) and its etiopathogenesis is still controversial (14). It has been related to nocturnal polyuria induced by hyposthenuria (13, 15, 16) and low functional bladder capacity (14, 17). Hyposthenuria is one of the earliest organic manifestations of SCD occurring as early as 12 months of age (13, 18). However, in a more recent study, Eneh et al. have shown that enuresis in children with SCD appears not to be related to hyposthenuria, but to other causal factors that apply to the general population (14).

The present cross-sectional study was conducted to estimate the prevalence of enuresis in individuals with SCD and its impact in the quality of life. We have hypothesized that enuresis is more prevalent in children, adolescents and young adults with SCD than described for general population, and that it greatly impacts the quality of life of this population.

## MATERIALS AND METHODS

This cross-sectional study was carried out from July 2016 to September 2017. During this period, 50 consecutive patients (children, adolescent, and young adults) with SCD, aged 5 to 24 years, who regularly attended the regional outpatient reference center for SCD, were evaluated. Patients with current urogenital disorder, current use of medications or diseases known to interfere with bladder or sphincter function, such as urinary tract infection and severe intellectual disability, and those not yet toilet trained were not included in the study.

**Figure 1:**
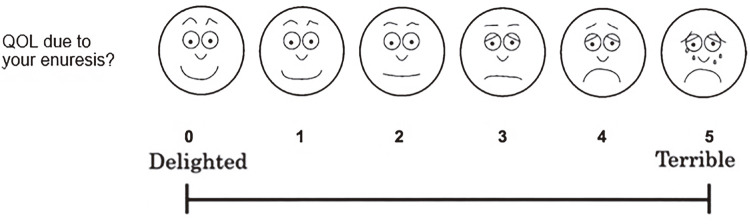
Visual Analog Scale. Visual Analogue Scale (VAS) adapted from Ushijima et al., 2006 VAS questionnaire to assess discomfort or satisfaction regarding patient’s quality of life (QOL). How would you rate your discomfort with enuresis? The face scales were demostrated to help understanding, includinf: 1) laughing face to represent delighted or pleased above left end of VAS, 2) smiling face to represent mostly satisfied above left side to center of VAS, 3) face with neutral expression to represent neither satisfied and dissatisfied above center of VAS, 4) face in trouble to represent mostly dissatisfied above righr side to center of VAS and 5) crying face to represent unhappy or terrible above right end of VAS.

A questionnaire was developed for this study and applied to the participants and/or their caregivers. Questions related to sociodemographic characteristics, hemoglobin electrophoresis for detect different hemoglobin genotypes, age of acquisition of complete toilet training, presence of LUTS and enuresis (including frequency and classification: monosymptomatic or non-monosymptomatic) were addressed. The Visual Analogue Scale (VAS), adapted from Ushijima et al., 2006 (19), with appropriate facial expressions throughout the VAS was used to analyze the quality of life. The VAS used in this study was a 10cm line ranging from delighted at the left end of the line up to terrible at the right end of the line. The subjects were asked to assign a score of 0 to 5 according to the intensity with which the enuresis affected their well-being with faces graphic expressing each note. The higher the score, the more uncomfortable the impact and the subject was in a situation (19, 20) (Figure-1).

Quantitative variables, continuous or ordinal, were described by measures of central tendency (mean or median) and the respective dispersion measures (standard deviation, interquartile range or minimum and maximum values). Nominal or qualitative variables were described for their absolute values, percentages or proportions. To compare the differences of continuous variables, we used the Student t-test or the Mann-Whitney test. For comparison of categorical data, we used the chi-square test and its variants. The association between the parameters studied was expressed by the prevalence ratio (Odds Ratio). A 95% confidence intervals were used as measures of precision of the results and p values less than 0.05 (p <0.05) were considered significant. In the analysis, we used computational statistical software (GraphPad Prism, version 8.0.0, GraphPad Software, San Diego-CA, USA).

The study was approved by the institution ethics committee, (CEP-UEFS #1.440.239) and all participants or legal representative that agreed in participate in the study signed a free and informed consent.

## RESULTS

In this study, a total of 50 patients with SCD were evaluated. The mean age at enrollment was of 10 years [7-15], being 52% (26/50) female. SCD genotypes diagnosed by hemoglobin electrophoresis at alkaline pH are shown in Table-1.

**Table 1:** General characteristics of the study population (n=50).

	Description	Percentage
Gender	Female	52 (26/50)
	Male	48 (24/50)
Age (years)	6-11	58(29/50)
	12-18	30(15/50)
	19-24	12(06/50)
Genotype	SS	58(28/50)
	SC	34(17/50)
	Sα-thalassemia	02(01/50)
	Undetermined	08 (04/50)

**Figure 2:**
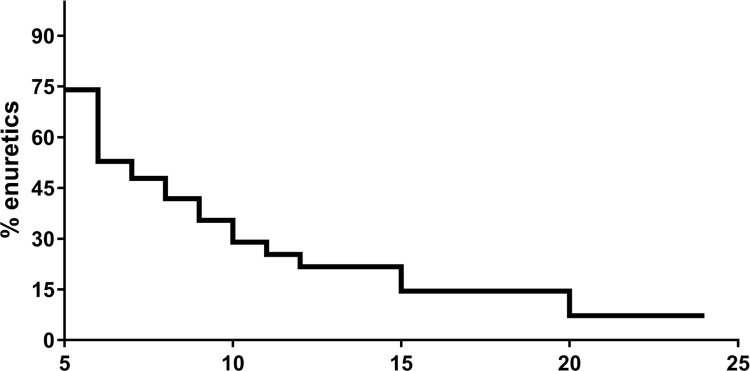
Prevalence of enuresis according to age.

Of the 50 individuals evaluated, enuresis was identified in 42% (21/50) of the cases, affecting 75% of the subjects at five years and about 15% of the adolescents at 15 years of age (Figure-2). Enuresis was classified as monosymptomatic in 33.3% (7/21) and non-monosymptomatic in 66.6% (14/21) of the cases, being primary in 100% of all subjects. In 62% of subjects frequency, enuresis episodes were more than three times a week. Nocturia was identified in 24% (12/50), urgency in 20% (10/50) and daytime incontinence 10% (5/50) of the individuals.

Enuresis appears to be more common in males (OR=1.89 [0.59-5.38]), but no statistical significance was found (p=0.395). Toilet training daytime was finished before completing two years of age in 32% (16/50) of patients, 24% (12/50) between two and three years, 20% (10/50) between three and four years, 14% (7/50) between four and five. The other five did not remember when toilet training was completed. No correlation was found between the age of completing toilet training daytime and the prevalence of enuresis.

Enuresis had a negative impact on the quality of life of those affected. Asymptomatic patients had significantly higher scores in VAS than symptomatic ones (9.26±0.92 and 7.22±3.44, respectively- p=0.015). According to VAS, 67% (14/21) of the study subjects considered that enuresis had a serious impact on their quality of life.

## DISCUSSION

The present study is in agreement with previous studies that have shown a significantly increase in the prevalence of enuresis in subjects with SCD (13, 15, 17, 21), as evidenced in 42% of the population studied herein. A recent study recruited 243 children with SCD aged between 5 and 17 years and showed a prevalence of 49.4% of enuresis versus 29.6% in the control group (22). The inclusion of patients as old as 24 years of age is justified as these studies have shown that enuresis remains prevalent in adolescents and young adult with SCD (12, 16, 22).

Although some studies have reported hyposthenuria as the main determinant of enuresis in individuals with sickle cell disease (23), recent studies have not shown this association (14).

In the present study, there was a higher prevalence of monosymptomatic enuresis (66%) and all cases were primary enuresis. This finding is in agreement with Portacarrero et al. who showed that children and adolescents with SCD had non-monosymptomatic and primary enuresis in 58% and 86% of the cases, respectively. Another recent study also demonstrated an increased prevalence of primary non-monosymptomatic enuresis in SCD patients (24).

It is important to note that 15% of adolescents at 15 years of age had enuresis. These results are also similar to those of Portacarrero et al. that demonstrated the prevalence of 21% of enuresis in adolescents with sickle cell disease (15-18 years of age) (12). They are also compatible with findings from Field et al. and Esezobor et al. who documented persistently high rates of enuresis of 18% and 25% among adolescents over 14 years of age, respectively (22, 25). On the contrary, studies in healthy population have shown that the prevalence of enuresis decreases with age to around 1 to 3% at 15 years (11, 26). Therefore, the well-established decline in the prevalence of enuresis with age was less pronounced in individuals with sickle cell disease.

Although Mabiala et al. documented a higher prevalence of enuresis in females with SCD (27), our findings are in agreement with the majority of other studies (12, 16, 22) that have found a higher prevalence of enuresis in males. The reason for this preponderance in males is still unclear. No correlation between age of acquisition diurnal urinary control and the presence of enuresis and other lower urinary tract symptoms, as in Down syndrome patients was found (7).

An increased prevalence of urgency and daytime incontinence was present in this series as well as in other series (15). Portacarrero et al. demonstrated 33% of urgency and 23% of daytime incontinence in SCD (12). In addition to these LUTS, 24% of subjects in the present study had nocturia. Enuresis and nocturia are common in children with SCD being reported to be present in 68 to 79% of the patients (15, 23, 25) Further prospective studies are needed to clarify the pathophysiological mechanisms underlying the urinary manifestations of SCD.

In this study, enuresis negatively affected the quality of life in 67% of the individuals. SCD is characterized by systemic complications including thoracic syndrome, chronic lung disease, cardiomyopathy, splenic infarction, chronic liver disease, bone infarction, osteomyelitis and depression (28, 29). All these complications, with prolonged hospitalizations and frequent chronic pain, significantly reduce quality of life (29, 30). Although quality of life of all patients included in the study may be compromised by SCD, those presenting enuresis had an even worse score when compared to non-enuretic ones, showing that enuresis negatively impact quality of life, as previously demonstrated by Savaser et al. for general population (11).

This study has several limitations small sample size and absence of a control group, family history of enuresis was not investigated, the main complications of SCD, which may have negative impact patient quality of life, have not been addressed and presence of intestinal constipation did not was evaluated.

## CONCLUSIONS

Enuresis was highly prevalent among children with SCD, and continues to be prevalent throughout early adulthood, especially in males generating a negative impact on quality of life. In all cases, enuresis was primary enuresis, and in the majority it was non-monosymptomatic. These findings are important to alert the parents and professionals involved in the follow-up of these patients about the need for diagnosing and treating this condition.
